# Moebius Syndrome: A Case Report on an Uncommon Congenital Syndrome

**DOI:** 10.7759/cureus.40746

**Published:** 2023-06-21

**Authors:** Ghizlane Souni, Ghanam Ayad, Aziza Elouali, Abdeladim Babakhouya, Maria Rkain

**Affiliations:** 1 Faculty of Medicine and Pharmacy, Mother and Child Health Laboratory, Mohammed I University of Oujda, Oujda, MAR; 2 Department of Pediatric Medicine, Centre Hospitalier Universitaire Mohammed VI, Oujda, MAR; 3 Department of Pediatrics, Faculty of Medicine and Pharmacy, Mohammed I University of Oujda, Oujda, MAR; 4 Department of Pediatric Gastroenterology, Centre Hospitalier Universitaire Mohammed VI, Oujda, MAR

**Keywords:** pediatric, pediatric population, nervus abducens paralysis, facial diplegia, moebius syndrome

## Abstract

Moebius syndrome (MS) is rare. It is defined by congenital bilateral paralysis of the sixth and seventh cranial nerves, resulting in an absence of mimicry and strabismus responsible for major relational disorders. Other cranial nerves can also be affected (third, fourth, fifth, ninth, tenth, and twelfth cranial pairs). In the majority of cases, MS is sporadic, causing problems with sucking, swallowing, breathing, and phonation. Associated malformations have also been reported. The disease is not progressive, and management is mainly symptomatic. We report a three-year-old girl who presented with facial asymmetry and in whom the MS was confirmed through magnetic resonance imaging (MRI). A multidisciplinary approach was conducted on our patient and is currently being followed up in the neuropediatrics department, and an ophthalmological examination is scheduled. Additionally, she had medical consultations with a plastic surgeon for smile rehabilitation. On the other hand, psychological support was maintained.

## Introduction

Moebius syndrome (MS) is an uncommon congenital syndrome distinguished by complete and bilateral facial paralysis, along with limited lateral movement of the eyes. It is defined by congenital bilateral involvement of the sixth and seventh cranial nerves, sometimes associated with other malformations [1]. Initially described in 1880 by von Graefe and Saemish, who, at that time, grouped certain patients with rare congenital disorders of the facial region that were nonprogressive, it was later validated in 1888 by Paul Julius Moebius, who named it *Moebius syndrome*. This syndrome is defined as congenital paralysis of the VI (abducens) and VII (facial) cranial nerves, as well as other cranial nerves such as the XII (hypoglossal) and III (oculomotor) nerves, with a variable clinical spectrum. Other cranial nerves may also be affected (V, X, and XI). Associations with multiple malformations such as limb malformations, especially Poland anomaly (insufficient pectoral muscles and a unique fold on the palm of the same side) or other hand and finger anomalies, mental retardation, and autism have been reported in some cases [1]. We report a three-year-old girl who was diagnosed with MS in its second pattern diagnosed through clinical asymmetry and confirmed by the absence of the left facial nerve on magnetic resonance imaging.

## Case presentation

We report a three-year-old girl, the youngest of three siblings, from a nonconsanguineous marriage. The pregnancy was uneventful, and regular checkups confirmed a healthy development. The pregnancy was carried to term without any incidents. No history of exposure to toxins or medications, including abortive products. The delivery was medically assisted by vaginal delivery with no history of trauma or forceps use, and good adaptation to extrauterine life with imprecise birth weight.

**Figure 1:**
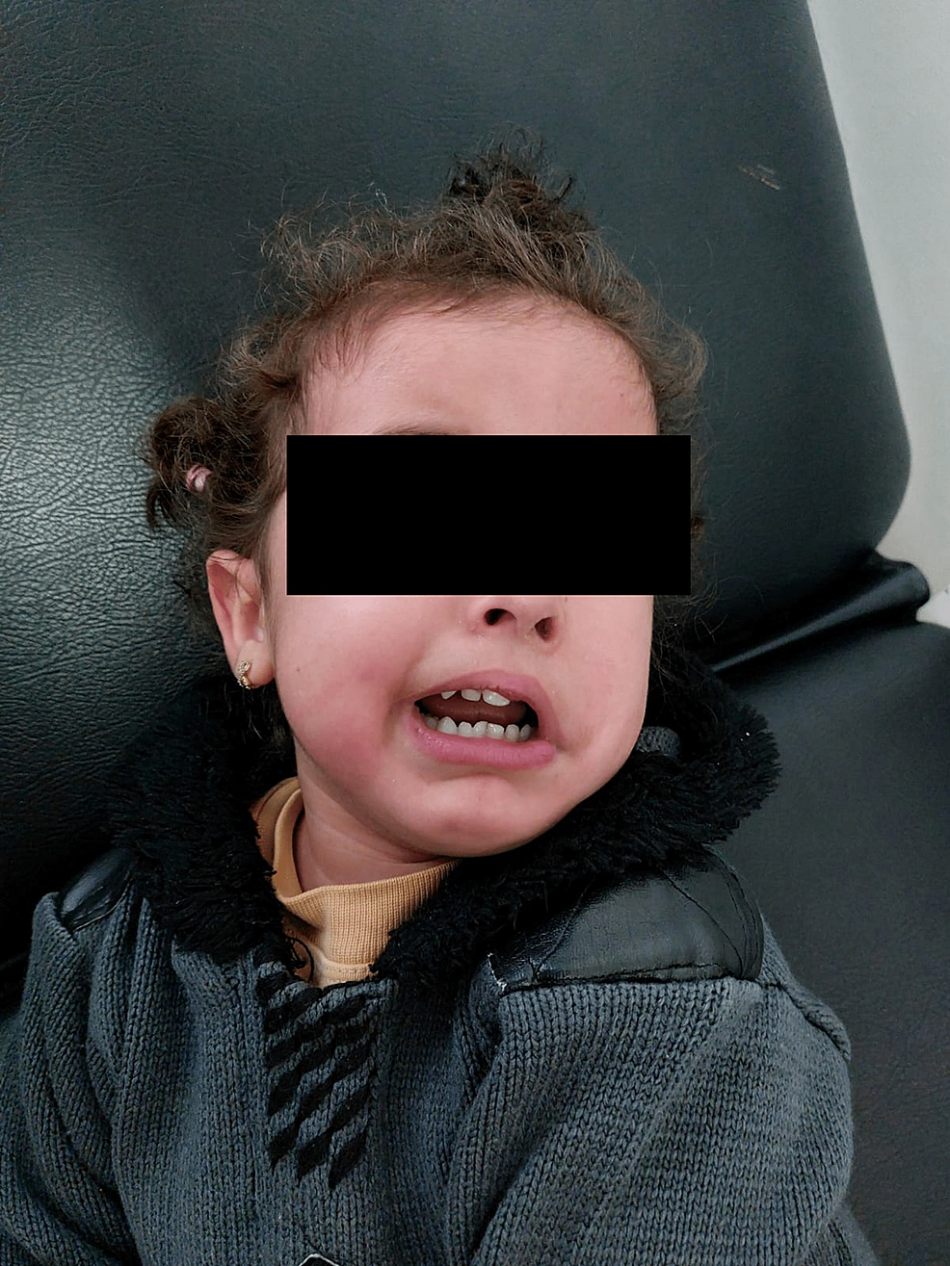
Our patient presents with left-sided facial paralysis.

The patient was brought to consultation for facial asymmetry at the age of 20 months. This asymmetry was, however, noticed by the mother when smiling since she was four months of age. The child had, up to this point, good psychomotor development with walking acquired at 12 months and speech at 18 months. On clinical examination, the patient aged six months showed a left-sided facial paralysis with deviation of the eye and lip, and effacement of the nasolabial fold was noted, which was more accentuated during crying. Mild convergent strabismus of the same was also noted. Furthermore, the patient did not present any facial dysmorphia or limb malformations (Figure 1).

A cerebral computed tomography scan was performed at six months of age, which returned normal; then the patient was lost to follow-up. Due to the lack of clinical improvement, the family sought further consultation, where magnetic resonance imaging (MRI) was requested, revealing the absence of visualization of the left facial nerve.

## Discussion

MS is a rare congenital condition caused by a disturbance of the rhombencephalon, with agenesis of the cranial nerves VII and VI (facial and abducens, respectively). The estimated prevalence of this condition is 1 in 125,000 live births, and it affects both sexes equally [2]. Although it appears to be genetic, its precise cause is still unknown [3]. In the vast majority of cases, it occurs sporadically without any family history or events during pregnancy [3]. In rare cases, inheritance has been observed, particularly in a dominant pattern, with some patients exhibiting expression in the form of Poland syndrome.

Four categories have been proposed based on physiopathological results that explain the physical manifestations and imaging findings. The first condition is marked by underdevelopment of the cranial nerve nuclei due to congenital abnormalities. The second condition, which our case belongs to, is distinguished by the degeneration and loss of neurons caused by a defect in the peripheral facial nerve. The third exhibits decreased neurons along with degeneration, gliosis, focal necrosis, and calcifications in the nuclei of the brainstem occurring as a result of either inadequate blood supply or infection. The last group displays primary myopathic changes without the involvement of the nuclei or cranial nerves.

Among these etiological causes, studies suggest the involvement of environmental and genetic factors, including exposure to teratogenic agents such as alcohol, cocaine, thalidomide, and misoprostol (a semisynthetic analog of prostaglandin E1 used to induce pregnancy termination that can cause in utero hypotension and other mechanisms such as hemorrhage, thrombosis, or stenosis involving the veins of the second pharyngeal arch, facial arteries, and failure of formation of the vertebrobasilar system triplets, thus affecting the development of cranial nuclei) during the first trimester of pregnancy, leading to vascular disruption of the developing brain.

One study identified mutations in the *PLXND1 *and *REV3L* genes [3]. The molecular basis of MS is diverse. Mederos-Mollineda et al. provided a comprehensive summary of the genetic and etiological literature underlying this disorder by selecting and examining articles from 1880 to 2013 to understand all molecular theories and the chronicity of literature progress, elucidate the genetic causes of the disease, and aid in early detection and treatment planning [1]. The results showed that mutations in the MBS1, MBS2, and MBS3 genetic loci contribute to the development of MS through various pathways. *HOX* genes from the family encoding homeobox domains were also involved in abnormal brain development [1]. The results of this study add to a growing database of mutations associated with this syndrome and can be used to diagnose MS and clarify its pathogenesis.

Although there is currently no prenatal test to determine MS, individuals can benefit from genetic counseling [1]. A classification was established by Terzis and Noah that distinguished between classic MS, where paralysis of both facial and abducens nerves is bilateral and complete; incomplete MS, which gives the clinical picture of the disease with some residual motor function on one side of the face; and Moebius-like syndrome where facial paralysis is unilateral and may be associated with involvement of other cranial nerves [4].

Clinically, facial nerve paralysis, which can be unilateral or bilateral, gives the impression of an amimic face with the presence of drooling, a smile may be absent, unattractive, or even grimacing due to the involvement of certain active subcutaneous muscle bundles [5]. Involvement of the sixth cranial nerve results in limitation of abduction, horizontal gaze paralysis, inability to perform saccadic movements, and optic nystagmus. Usually, there is no paralysis of vertical gaze in the syndrome, but it can occur in up to 25% of cases with the involvement of the third pair [3]. In a study conducted in Mexico focusing on ophthalmological involvement in this syndrome, the highest percentage was found in limited ocular sequestration and facial paralysis (100%), esotropia (54%), epicanthus (51.5%), entropion (22%), and history of abortion-inducing agents used by the mother during the first trimester of pregnancy (28%). Hypertropia and exotropia were also found in some cases, concluding that MS has diverse ophthalmological manifestations that need to be detected early to improve function and aesthetics [1]. MS can also involve other anomalies, including damage to other cranial nerves such as the hypoglossal nerve (30%), causing language and speech disorders and drooling, the trigeminal nerve (8%), the oculomotor nerve and, less commonly, the glossopharyngeal nerve. It can also be associated with a malformation syndrome, including micrognathia, cleft palate, heart malformations (such as Taussig-Bing anomaly), urinary and orthopedic anomalies (such as clubfoot), and digital malformations of the upper limbs (syndactyly).

MS can be detected during the early stages of infancy. It is characterized by various symptoms such as inadequate lip closure leading to difficulties in sucking, lack of facial expressions, incomplete eyelid closure during sleep, excessive salivation, and inward deviation of the eyes (esotropia). Newborns affected by this condition commonly experience challenges with swallowing and breathing. Moreover, around 90% of these individuals exhibit craniofacial malformations that increase the likelihood of airway blockage in newborns. Cuestas et al. conducted a study in 2019 on seven newborns with this syndrome; they found that all patients were affected by strabismus and bilateral facial paralysis, 42.9% had a reported maternal history of utilizing misoprostol during the initial trimester of pregnancy as a means to induce abortion, 42.9% had associated malformations, including Poland syndrome in one newborn (hypoplasia of the major pectoral muscle); and Pierre-Robin sequence in two patients (retrognathia, cleft palate, and glossoptosis). The examination detected involvement of other cranial nerves in four newborns, six of them were hypotonic, two had heart malformations, four had thoracic and external ear malformations, and all had a clubfoot. In addition, micrognathia, palatal anomalies, and glossoptosis were present in 85.7%, and retrognathia in 42.9%. Three individuals experienced severe respiratory distress, necessitating intubation initially and subsequently undergoing a tracheotomy. All patients were fitted with nasogastric tubes shortly after birth and received treatment for reflux along with early interventions for improving swallowing abilities. At a more advanced stage of life, this syndrome can have a psychological and social impact. In a set of focus group investigations, teenagers diagnosed with MS expressed that the condition carried a significant stigma, primarily resulting from its rarity, facial distinctiveness, and absence of facial expressions. Furthermore, individuals with MS encounter challenges in being properly understood by others due to misinterpretation of their speech and general unawareness regarding this syndrome. MS remains a visible yet underrecognized condition. Owing to their limited facial expressiveness, people with MS are frequently mistaken for unfriendly, sorrowful, or intellectually impaired individuals. The diagnosis of this syndrome is mainly clinical, identifying neurological damage, with imaging results confirming hypoplasia or absence of cranial nerves, especially VI and VII, calcifications in the lower brainstem, or hypoplasia of the cerebellum and brainstem [3]. The management of children with SM requires a multidisciplinary team. Respiratory assistance or even tracheotomy may be necessary for the neonatal period due to respiratory difficulties that may endanger the patient's life. Many children require numerous surgeries, including facial reconstruction, surgical correction of strabismus, or orthopedic surgery. Similarly, early rehabilitation is crucial to optimize the recovery of various altered functions and improve the outcomes and quality of life of these patients [2].

## Conclusions

The diagnosis of MS must consider all ophthalmological and general signs, and it can be difficult in some atypical cases. The management of these patients should be provided by a multidisciplinary team. There are very few reports on the syndrome, and genetic counseling in these cases is still debated, given the heterogeneity of the disease's origin.
